# Increasing melanism along a latitudinal gradient in a widespread amphibian: local adaptation, ontogenic or environmental plasticity?

**DOI:** 10.1186/1471-2148-10-317

**Published:** 2010-10-21

**Authors:** Jussi S Alho, Gábor Herczeg, Fredrik Söderman, Anssi Laurila, K Ingemar Jönsson, Juha Merilä

**Affiliations:** 1 Ecological Genetics Research Unit, Department of Biosciences, PO Box 65, FI-00014 University of Helsinki, Finland; 2 Population and Conservation Biology, Department of Ecology and Evolution, Evolutionary Biology Centre, Uppsala University, Norbyvägen 18D, SE-75236 Uppsala, Sweden; 3 School of Teacher Education, Kristianstad University, SE-29188 Kristianstad, Sweden; 4 Department of Genetics, Microbiology & Toxicology, Stockholm University, SE-10691 Stockholm, Sweden

## Abstract

**Background:**

The thermal benefits of melanism in ectothermic animals are widely recognized, but relatively little is known about population differentiation in the degree of melanism along thermal gradients, and the relative contributions of genetic *vs. *environmental components into the level of melanism expressed. We investigated variation in the degree of melanism in the common frog (*Rana temporaria*; an active heliotherm thermoregulator) by comparing the degree of melanism (i) among twelve populations spanning over 1500 km long latitudinal gradient across the Scandinavian Peninsula and (ii) between two populations from latitudinal extremes subjected to larval temperature treatments in a common garden experiment.

**Results:**

We found that the degree of melanism increased steeply in the wild as a function of latitude. Comparison of the degree of population differentiation in melanism (*P_ST_*) and neutral marker loci (*F_ST_*) revealed that the *P_ST _* >*F_ST_*, indicating that the differences cannot be explained by random genetic drift alone. However, the latitudinal trend observed in the wild was not present in the common garden data, suggesting that the cline in nature is not attributable to direct genetic differences.

**Conclusions:**

As straightforward local adaptation can be ruled out, the observed trend is likely to result from environment-driven phenotypic plasticity or ontogenetic plasticity coupled with population differences in age structure. In general, our results provide an example how phenotypic plasticity or even plain ontogeny can drive latitudinal clines and result in patterns perfectly matching the genetic differences expected under adaptive hypotheses.

## Background

Melanins and carotenoids are two classes of pigment compounds that are responsible for much of the variation in animal coloration [1]. Melanins have been shown or suggested to be involved in a wide range of vital adaptive functions in animals, including signaling [2,3], crypsis [4,5], thermoregulation [6-10], protection from ultraviolet radiation [11,12] and immune function [13]. Whether melanism has a significant role in ectotherm thermoregulation has been subject to a lot of research over many decades [14], and the thermoregulatory explanation has been typically favored over other hypotheses. However, while the hypothesis about the thermally adaptive value of melanism has a lot of intuitive appeal because of its simplicity, the relationship between phenotype and genotype is complex [15-17], and other adaptive and non-adaptive causes should also be considered. Further, even if melanism in some circumstances is thermally adaptive, the question of whether it is a purely genetic or environmentally driven plastic trait is important, especially in light of the recent rapid anthropogenic changes in the environment [16]. Namely, changes in degree of melanism in a population could be driven by direct environmental induction (i.e. plastic response) as well as by selection acting on heritable genetic variation (i.e. adaptation).

Intraspecific variation in melanism, when present, can express itself as distinct color morphs including completely dark, or melanistic, individuals [6,18,19], or as a continuum of animals with different amounts of dark pigmentation [20,21]. The possible genetic basis of this variation has received considerable attention in literature. Several researchers have found significant family effects in common garden experiments, raising the possibility of genetic variation in the degree of melanization [14,22]. There are also numerous molecular genetic studies that have investigated the association between melanism and sequence variation e.g. in the melanocortin-1 receptor gene [23-27] and its antagonist, the agouti signaling protein (*agouti*) [28,29]. These molecular analyses have often, although not always, found a correspondence between certain genotypes and phenotypes. However, few studies have attempted to establish an adaptive basis for variation in melanization by linking the genetic variation to pigmentation clines observed in nature (but see e.g. [25,30-33]).

Since melanism is genetically based in numerous taxa [5,23,24,26] and has been shown to confer thermally adaptive advantage [6,8-10], one would expect to observe intraspecific pigmentation clines along latitudinal or altitudinal gradients in the wild, as these gradients typically correspond to ambient temperature. However, such clines have been rarely reported in other taxa than insects [14] and the genetic basis, and thus the adaptive nature, of melanism clines remain little explored. To show that melanism can be thermally adaptive, one needs to simultaneously demonstrate both the existence and the genetic basis of a melanism cline along an environmental gradient. A failure to find genetic basis for an observed trend would indicate that the cline is caused either by environmentally induced plasticity (which can itself be either adaptive or non-adaptive), or by ontogenetic plasticity coupled with geographical differences in age or longevity. For instance, given that the degree of melanism in at least some anuran amphibians increases with age [20], and average age of individuals in a given population tends to increase with increasing latitude and altitude [34,35], changes in population age structure could also be driving clines in the degree of dark pigmentation.

Here, we studied the existence and possible genetic basis of a latitudinal cline in the degree of melanization in the common frog (*Rana temporaria*). Amphibians are a particularly well-suited group of organisms to study the genetic basis of pigmentation due to the large color variation they exhibit within and between species [22,36]. The common frog makes an especially interesting model since (i) it has one of the widest distribution ranges among amphibians including both high altitudes and latitudes [37], (ii) it is an active heliotherm thermoregulator [10], (iii) exhibits variation in the level of melanization [10,27], and (iv) the degree of melanization in the species has been indicated to correlate positively with heating rate [10]. Furthermore, many traits in this species have previously been shown to have undergone adaptive divergence along a latitudinal gradient [38-41].

The aims of our study were twofold. First, we investigated the presence of a latitudinal trend in the degree of melanization in wild-caught adult common frogs. Second, we used a common garden experiment to test whether the observed cline in melanism was genetically based, or reflected either plastic response to environmental heterogeneity or ontogenetic plasticity with geographical differences in age structure. To these ends, we compared the level of dorsal melanism in twelve populations along over 1500 km latitudinal gradient across the Scandinavian Peninsula, and conducted a common garden study applying larval thermal treatments using two latitudinally extreme populations. We hypothesized that a latitudinally ordered thermal adaptation might have occurred in *Rana temporaria *favouring elevated heating rates towards higher latitudes. Based on this hypothesis we predicted that the level of melanism would increase towards north and that the pattern would at least partially be genetically based and independent of rearing environment.

## Methods

### Study species

**Figure 1:**
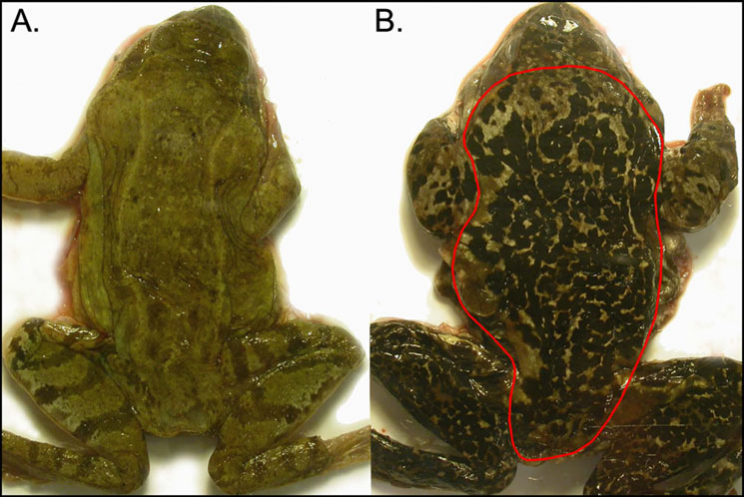
**Representative photographs of light (a) and melanistic (b) wild adult common frogs**. The images illustrate the variation in the degree of melanism, and the red outline the dorsal area from which the proportion of melanistic area was measured.

The common frog is the most widely distributed anuran amphibian in Europe: it can be encountered from Spain to the Ural Mountains in Russia, from sea level to altitudes above 2000 m [37]. In the north it reaches the North Cape in Norway. The coloration of the adult frogs varies from reddish brown to olive green, and many individuals have varying degree of distinct black markings on their dorsal surface (Figure 1) [27,42]. The visible color has two components: the underlying 'base' color that the frogs can alter relatively quickly, and the color caused by the dark melanistic spots which were the focus of this study [27]. Studies of intrapopulation variation in this species have revealed that the amount of black on dorsal surface can be highly variable within a single population [10], and the trait shows ontogenetic variability [20]. Since the degree of melanistic patterning influences heating rates, it has been hypothesized that the amount of black dorsal pigmentation could have adaptive value allowing more efficient thermoregulation in a cold climate [10]. Collection of adult frogs and eggs was done with the permissions from national and regional authorities and the common garden experiment was conducted with the permission C194/6 from the Ethical Committee for Animal Experiments in Uppsala County in accordance with national and international guidelines.

### Data from the wild

**Figure 2:**
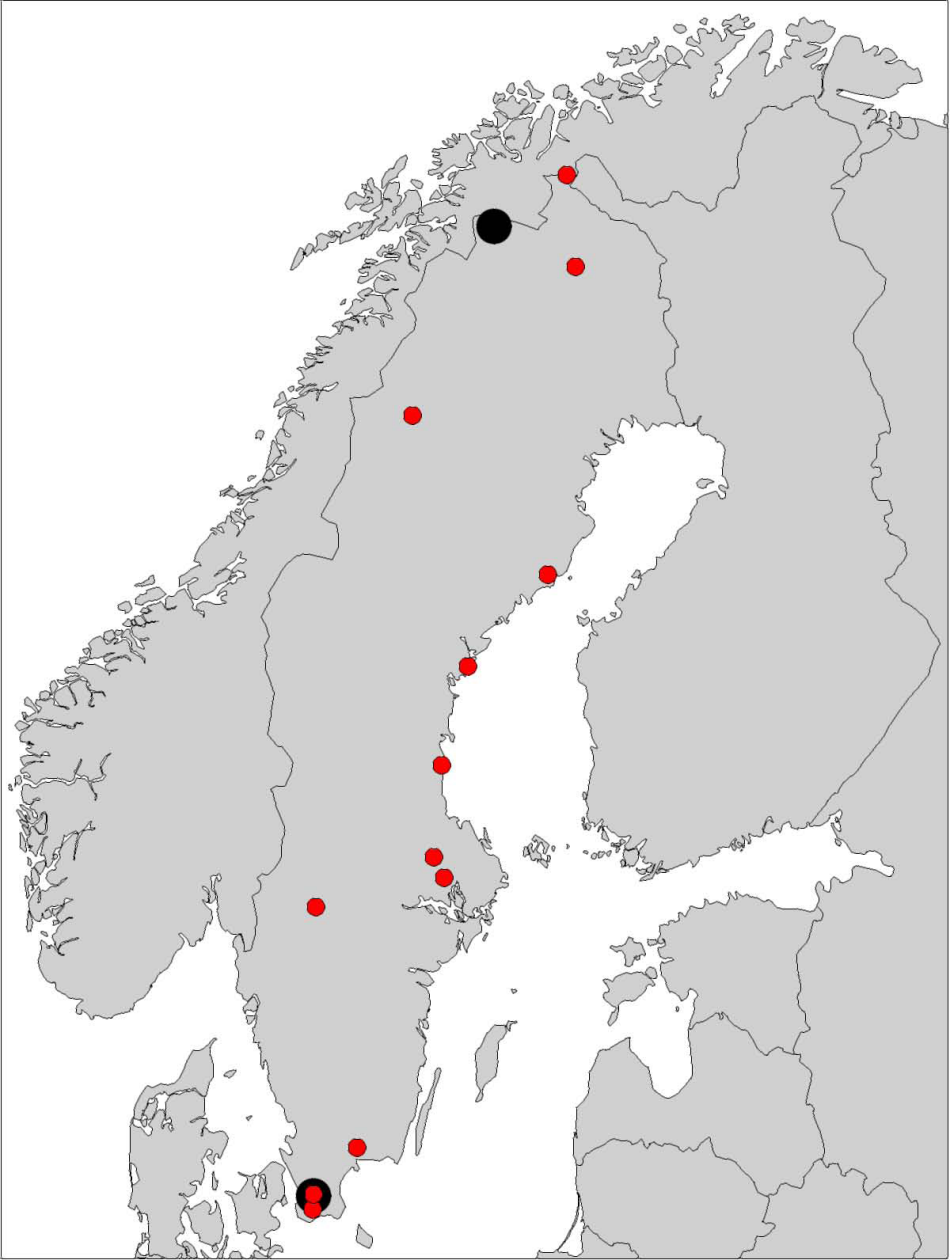
**Map showing the locations of the study populations in Sweden and Finland**. Data from the wild originated from the populations marked by red circles, and populations used in the common garden experiment are marked with larger, black circles.

Data on wild adults was collected during the breeding seasons of 1998-1999 as part of other studies [38,40,41,43] from twelve localities (Figure 2, Table 1). In short, a total of 111 adult female and 113 male common frogs were collected during the early breeding season, right after emergence from hibernation (April-June, depending on latitude). Live frogs were transported to laboratories in Uppsala or Lund where they were anesthetized and killed with an overdose of MS-222 (tricaine methanesulfonate). Each individual was sexed on the basis of gonadal inspection. Snout-vent length was measured with dial calipers to the nearest millimeter. Frog carcasses were frozen in -20°C until scored for the degree of melanism (see below).

**Table 1:** The locations of the study populations and sample sizes (N) in the analyses

Wild data			
		**N**	
		
**Population**	**Coordinates**	**Females**	**Males**

Börringe, Svartesjöhus	55° 30' N, 13° 25' E	7	13
Revinge, Tvedöra	55° 42' N, 13° 26' E	10	9
Blekinge, Hemsjö	56° 19' N, 14° 42' E	10	10
Karlstad, Lindrågen	59° 28' N, 13° 31' E	11	8
Järlåsa, Häggedal	59° 51' N, 17° 14' E	9	9
Tärnsjö, Gullsmyra	60° 07' N, 16° 56' E	9	9
Söderhamn	61° 16' N, 17° 11' E	10	9
Härnösand	62° 37' N, 17° 59' E	10	10
Umeå, Grytan	63° 49' N, 20° 14' E	8	10
Ammarnäs	65° 54' N, 16° 18' E	10	10
Kiruna, Esrange	67° 51' N, 21° 02' E	10	10
Kilpisjärvi, Malla	69° 03' N, 20° 47' E	7	6
Total		111	113

**Common garden data**			

		**N**	
		
**Population**	**Coordinates**	**Families**	**Offspring**

Revinge, Tvedöra	55° 42' N, 13° 26' E	15	32
Abisko, Björkliden	68° 24' N, 18° 40' E	20	139
Total		35	171

### Common garden data

A common garden experiment was conducted for two populations along the latitudinal gradient across the Scandinavian Peninsula (Figure 2, Table 1). In the case of the southern population adult frogs were collected from spawning sites and kept in 4 °C until used in artificial fertilizations. Crosses were performed following North Carolina type I design [44]. Briefly, the experiment utilized twenty females and ten males from the southern population, and each male was crossed with two females. This resulted in twenty full-sib and ten paternal half-sib families. Artificial fertilization was performed following [45] with some modifications. Males were first injected with hormone, the cloaca was rinsed with Ringer's solution and the sperm solution was separated into two containers. The eggs were squeezed out from females' cloaca to the sperm solution with the help of blunt forceps. In the case of the northern population we collected eggs from the wild. These naturally laid eggs were subsequently handled in similar manner to those from artificial crosses after fertilization.

The eggs and hatchlings from each full-sib family were raised in two 3 L vials (ca. 200 eggs in each) until Gosner stage 25 [46]. Temperature in the laboratory room was 18 °C and the light rhythm 18L:6D. After reaching stage 25, ten tadpoles from each cross were raised individually in 1 L vials at two temperature treatments, 18 °C (high) and 14 °C (low), in two separate climate-controlled rooms with 18L:6D light rhythm. There were thus five tadpoles per cross per treatment. During this time the tadpoles were fed slightly boiled spinach *ad libitum*. To assure homogenous water quality, the animals were reared throughout the aquatic stage in reconstituted soft water (RSW) [47] renewed every third day. After metamorphosis, the tadpoles were moved to 1 L boxes with the bottom covered with moss and with a dripping system maintaining moisture in a climate room maintained at 16 °C. At this stage, the juvenile frogs were fed with unlimited amount of *Drosophila *flies and small crickets. In late November, the juveniles were moved to another climate chamber where the conditions were gradually changed to resemble wintering conditions (0L:24D, 4 °C). They were allowed to overwinter until late February, when the conditions were gradually changed to correspond to the summer conditions again. The juveniles were then maintained under similar conditions as above. Nine months after metamorphosis, all juveniles were photographed for the estimation of the degree of melanism (note that this date varies between individuals due to the difference in phenology between the populations and in development rate between the larval temperature treatments). Snout-vent length of the juveniles was measured from the digital photographs.

Mortality limited the available data in the southernmost population mostly to full-sib families. Since also the data on the northernmost population came from full-sib families, we restricted our analysis below to family effects, and did not attempt to estimate sire and dam effects.

### Estimation of level of melanism

Estimation of the relative area covered by the permanent melanistic spots was done similarly with the wild-caught and common garden samples. The individuals' (thawed carcasses in the wild-caught and live specimens in the common garden sample) dorsal sides were gently pressed against a transparent plexiglass panel to obtain a nearly flat surface for photographing. Digital photographs were taken under similar circumstances (different sets for wild-caught and common garden samples). A ruler or millimeter paper was placed in every photograph for scaling. Color calibration was not employed, but melanistic and non-melanistic areas were clearly distinguishable in the images, as shown by the relatively high repeatability estimate for the degree of melanism (see Results).

The digital photographs were processed with Image Pro Plus 4.5 software (Media Cybernetics Inc., Bethesda, USA). We first defined the area (the dorsal torso without head and appendages; Figure 1b) and then the color of the spots of interest. Then, with the aid of the scale on each photograph, the software automatically calculated the area of each spot and the remaining area. The degree of melanism was defined as the proportion of dorsal surface area covered by pigmentation spots larger than 1 mm^2^. 24 images were measured twice in order to estimate the repeatability for length and the degree of melanism.

### Statistical analyses

Since the degree of melanism was a proportion, it was arcsine transformed for the statistical analyses, i.e. we calculated the arcsine of the square root of the proportion. Back-transformation, when needed, was done by taking the square of the sine of the value.

We calculated the repeatability of snout-vent length and the degree of melanism for the juveniles reared in the common garden following [48]. In short, one-way analysis of variance using the functions *lm *and *anova *in R [49] was used and the repeatability was derived as:

(1) $$r=\frac{s_{A}^{2}}{s^{2}+s_{A}^{2}}$$

where *s*^2 ^was the within individuals mean squares and $s_{A}^{2}$ was calculated from

(2) $$s_{A}^{2}=\frac{MS_{A}-MS_{W}}{n}$$

where *MS_A _*was the among individuals mean squares, *MS_W _*the within individuals means squares, and *n *the number of measurements per individual, i.e. two. 95% confidence intervals for the repeatability estimates were obtained by non-parametric bootstrap, resampling the data 5000 times.

Latitudinal trends in the degree of melanism in adults caught from the wild were tested by fitting a linear mixed model using the *lmer *function of the *lme4 *package in R. The *lmer *function used restricted maximum likelihood (REML) approach. Sex, latitude, snout-vent length (used as a rough proxy for age), the interaction of sex and latitude, and the interaction of sex and snout-vent length were included as fixed effects, and population as a random effect. The model was fitted to the arcsine transformed data and predictions of the effect of latitude and snout-vent length were back-transformed for purposes of visualization. 95% highest posterior density intervals (HPDI) obtained with the functions *mcmcsamp *and *HPDinterval *of the *lme4 *package were used as a confidence measure. We also calculated the degree of phenotypic divergence (*P_ST_*) [50] between the populations as:

(3) $$P_{ST}=\frac{\sigma_{GB}^{2}}{\sigma_{GB}^{2}+2h^{2}\sigma_{GW}^{2}}$$

where $\sigma_{GB}^{2}$ was the variance between populations, $\sigma_{GW}^{2}$ the variance within populations, and *h*^2 ^the heritability. *P_ST _*was estimated for two scenarios, with heritability values *h^2 ^*= 1 and *h^2 ^*= 0.5. The variance components were estimated using the *lmer *function. We fitted a linear mixed model with sex and snout-vent length as fixed effects and population as a random effect. 95% confidence intervals for the *P_ST _*estimates were obtained by non-parametric bootstrap, resampling data 5000 times.

An estimate of *F_ST _*- describing the degree of neutral genetic divergence [51,52] - calculated based on six of our twelve study populations was available from a previous study [39]. We calculated *P_ST _*separately both for the six populations and for all twelve populations.

We investigated the population (i.e. among population additive genetic) effects in the degree of melanism in metamorphosed juveniles reared in the common garden setting. We again fitted a linear mixed model in R using the *lmer *function of the *lme4 *package. Population, snout-vent length, larval temperature treatment, and the interaction of population and larval temperature treatment were included as fixed effects and family as a random effect. The model was fitted to the arcsine transformed data. 95% highest posterior density intervals were again used as a confidence measure. The inclusion of family effect to the model corrected for the non-independence of full-sib measurements, and allowed us to estimate the upper limit for heritability *h^2 ^*[44] for the extremity length as:

(4) $$h^{2}\leq \frac{2\sigma_{F}^{2}}{\sigma_{F}^{2}+\sigma_{R}^{2}}$$

where $\sigma_{F}^{2}$ was the family variance and $\sigma_{R}^{2}$ residual variance. Family and residual variances were assumed to be equal between the two populations. 95% confidence interval for the heritability estimate was obtained by non-parametric bootstrap, resampling family data 5000 times.

## Results

The repeatability for the juveniles reared in common garden was 1.00 (F_23,24 _= 427.26; 95% CI: 0.98-1.00) for snout-vent length and 0.81 (F_23,24 _= 9.35; 95% CI: 0.25-1.00) for the degree of melanism. The snout-vent length was 16.9-27.1 mm in the common garden juveniles and 53.3-90.6 mm in the adults from the wild.

**Figure 3:**
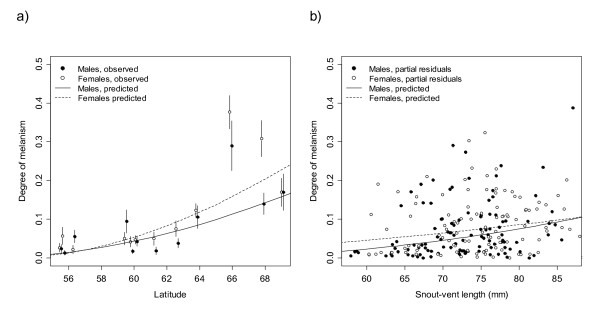
**The effects of latitude and body length on the degree of melanism**. The figure shows the relationship between a) latitude and the degree of melanism and b) snout-vent length and the degree of melanism in adult common frogs caught from the wild. The degree of melanism is defined as the proportion of dorsal surface area covered by pigmentation spots large than 1 mm^2^. In figure (a) the circles represent means and vertical bars standard errors of the means. In figure (b) the circles represent partial residuals after correcting for population effects and latitude (fixed at the mean of the data, 61°16'N). The curves in both figures describe the predicted degree of melanism. The prediction curves and partial residuals are based on a linear mixed model incorporating sex, latitude, snout-vent length, the interaction of sex and latitude, and the interaction of sex and snout-vent length as fixed effects, and population as a random effect. The model was fitted to arcsine transformed data. The curves are back-transformed predictions and hence not linear.

We found an increasing latitudinal trend in the degree of melanism in the adults caught from the wild, with suggestive sex differences so that melanism in females increased more steeply with latitude than in males (Figure 3a, Table 2). The snout-vent length had a weak positive effect on the degree of melanism, but there was no significant interaction effect of sex and snout-vent length (Figure 3b, Table 2). The degree of phenotypic divergence, *P_ST_*, for all twelve wild populations was 0.36 (95% CI: 0.26-0.45) assuming *h^2 ^*= 1, and 0.53 (95% CI: 0.43-0.63) assuming *h^2 ^*= 0.5. *P_ST _*for the six populations for which the degree of neutral marker divergence, *F_ST_*, was available, was 0.42 (95% CI: 0.32-0.52) for *h^2 ^*= 1, and 0.59 (95% CI: 0.48-0.69) for *h^2 ^*= 0.5. *F_ST _*published in [39] for these populations was 0.24 (95% HPDI: 0.18-0.30).

**Table 2:** Fixed effect estimates for the degree of melanism in wild-caught adult common frogs

			95% HPDI	
	
Effect	Estimate	SE	Lower	Upper
Intercept	-1.875	0.387	-2.587	-1.161
Sex	0.325	0.291	-0.250	0.900
Latitude	0.030	0.007	0.018	0.042
Latitude × Sex	-0.009	0.005	-0.018	0.001
Snout-vent length	0.004	0.002	-0.000	0.009
Snout-vent length × Sex	0.002	0.003	-0.004	0.008

The data was arcsine transformed. The model was a linear mixed model incorporating population as a random effect. Females were used as the reference sex. SE = standard error, 95% HPDI = highest posterior density interval.

**Figure 4:**
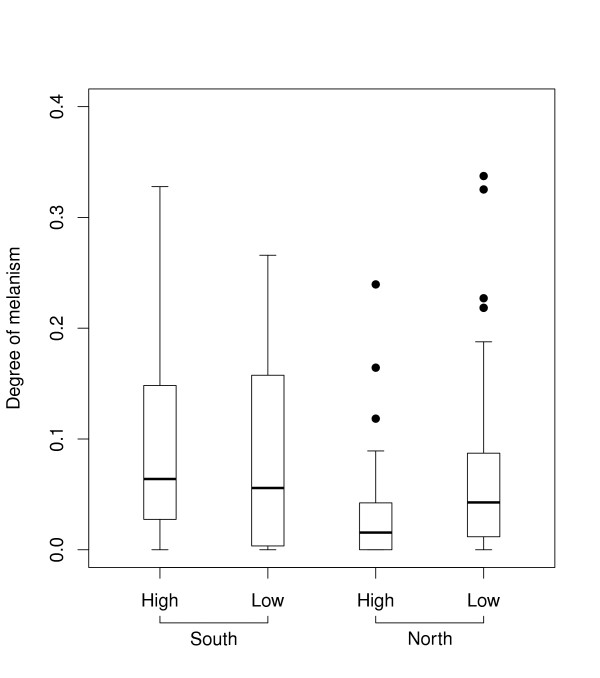
**Degree of melanism in juvenile common frogs**. Boxplot of the raw data from two populations ('North' = Björkliden, 68° 24' N; and 'South' = Tvedöra, 55° 42' N) reared in common garden. The degree of melanism is defined as the proportion of dorsal surface area covered by pigmentation spots large than 1 mm^2^. The boxes extend from 25th to 75th percentile, and the horizontal bands within the boxes are the medians. The whiskers represent the lowest and highest observation within 1.5 times the interquartile range from the lower and higher quartile, respectively. Labels 'High' and 'Low' describe temperature treatments.

Snout-vent length did not have a significant effect on the degree of melanism in juveniles reared in common garden (Table 3). There was a significant population difference in melanism suggesting additive genetic variation among populations (Figure 4, Table 3), but it was in the opposite direction than the latitudinal trend in the wild. The difference back-transformed from the effect estimates (Table 3) to difference in the degree of melanism between northern and southern population in the high larval temperature treatment corresponded to 9.8 percentage points. Cold larval temperature treatment increased the degree of melanism among the juveniles originating from the northern population (Figure 4, Table 3). In these, the difference in the degree of melanism between temperature treatments corresponded to 4.8 percentage points. The estimated upper bound for the heritability of melanism was 0.21, but the confidence intervals were wide (95% CI: 0.00-0.53).

**Table 3:** Fixed effect estimates for degree of melanism in juvenile common frogs reared in common garden

			95% HPDI	
Effect	Estimate	SE	Lower	Upper
Intercept	0.230	0.177	-0.136	0.569
Snout-vent length	-0.005	0.008	-0.021	0.013
Population	0.167	0.066	0.041	0.298
Temperature	0.092	0.030	0.027	0.146
Population × Temperature	-0.128	0.030	-0.275	-0.001

The data was arcsine transformed. The model was a linear mixed model incorporating family as a random effect. There were two temperature treatments, 'Warm' (used as a reference) and 'Cold', and two populations, 'North' = Björkliden (68°24'N; used as a reference) and 'South' = Tvedöra (55°42'N). SE = standard error, 95% HPDI = highest posterior density interval.

## Discussion

The thermoregulation hypothesis, stating that dark individuals have a fitness advantage in low temperatures as compared to light individuals due to their ability to heat up faster [14], is perhaps the most prominent adaptive explanation for melanism in ectotherms. However, few studies have tested whether the pigmentation clines observed in nature and explained as thermal adaptations are genetically based as assumed by the adaptive hypothesis (but see e.g. [30,32,33]). Here we detected an increasing latitudinal trend across the Scandinavian Peninsula in the degree of melanism in common frogs, but found no evidence of corresponding genetic differentiation in a common garden experiment with different larval temperature treatments. In fact, the observed genetic differences between populations were in the opposite direction of the latitudinal trend observed in the wild. As the evidence thus points against direct local adaptation as an explanation for the patterns we found in the wild, it seems plausible that environmental plasticity and/or ontogenetic plasticity together with geographic differences in age structure are responsible for the observed latitudinal trend. Interestingly, the trend persisted even after correcting for differences in snout-vent length - a rough proxy for age [53] - suggesting that the cline in nature could be more than a simple reflection of differences in age structure. This interpretation was supported by a significant environmental effect in the common garden experiment, with the juveniles originating from the northern population exhibiting higher degree of melanism in the colder larval rearing environment (Table 3). While previous studies on amphibians have found that both larval and adult coloration is affected by temperature and developmental stage [54-56], this is, to our knowledge, the first to suggest that the effects of larval environment on coloration can be carried over to the terrestrial stage. In light of these results, it is not necessarily surprising that an earlier study [27] did not find an association between MC1R sequence variation and variation in the degree of melanism in our study species.

Comparison of quantitative and neutral marker differentiation, as measured by *Q_ST _*and *F_ST_*, respectively, is often used to infer the relative roles of natural selection and random drift in among-population divergence [51,52]. The argument is that when *Q_ST _*exceeds *F_ST _*there is evidence of divergent selection, when the reverse is true there is support for stabilizing selection, and when *Q_ST _*and *F_ST _*are not significantly different, random genetic drift as the only cause of phenotypic divergence cannot be excluded [51]. When *Q_ST _*estimates have not been available, *P_ST _*values calculated from purely phenotypic data have been used as surrogates [50,57]. In our study, the degree of phenotypic divergence, measured as *P_ST_*, was significantly higher than the *F_ST _*estimate published in [39] for our populations, when the more realistic *P_ST _*value estimated assuming *h^2 ^*= 0.5 was used. Hence, looking naïvely at the phenotypic trend and divergence, without a further genetic analysis or study of population demography, the results suggest that the populations might have diverged under natural selection consistent with thermal adaptation hypothesis of melanism. However, when looking at the genetic population effects, not only do we find that there is no direct genetic basis for the latitudinal trend, but also that the observed population effects are to the opposite direction of the cline. Our results are thus in line with the criticism of the use of *P_ST _*in studies of wild populations [58].

The problem of disentangling genetic and environmental effects in studies of geographic or temporal phenotypic variation is a general one [16,59,60]. The relevance of this issue is likely to increase with ongoing rapid anthropogenic changes in the environment and the increasing integration of evolutionary biology to nature conservation in predicting the long-term effects of these changes [16]. Our study cautions once again against interpreting phenotypic trends - even if making perfect evolutionary sense - as evidence of local adaptation without explicit genetic analyses. In other words, environmental plasticity or even plain ontogeny can produce patterns that conform precisely to those expected under adaptive hypotheses (see also e.g. [59,61,62]).

Environmental plasticity itself can be either adaptive or non-adaptive [63]. Although somewhat speculative with only two populations, it is interesting to note that while larval rearing temperature had a significant effect on the degree of melanism in the common garden juveniles in the northern population (Table 3), there was also a significant interaction between population and temperature, with the southern population predicted to reverse the pattern observed in the north. This difference suggests that if melanism serves a thermoregulatory function in the north, natural selection might have acted on environmental plasticity, causing the individuals in the north to respond to cold temperature with increased melanistic coloration. However, the limited number of populations and the lack of accurate age data from the wild prevent any firm conclusions. The observed pattern in the wild might also reflect plain age differences or adaptive environmental plasticity serving other functions, such as protection from ultraviolet radiation [12], crypsis [4,5], disease resistance [13], or multiple simultaneous functions [14]. Finally, the pattern might arise from a correlation with an adaptive trait, or from entirely non-adaptive environmental plasticity [63]. Without measuring age and selection, the adaptive significance of the degree of melanism in the common frog is, however, speculative and the realm of future studies.

Our study comes with a number of caveats. The sample size in the common garden data was small in terms of populations, families and offspring. This was reflected e.g. in the fact that the confidence interval for the upper limit for heritability was very wide and prohibited any firm conclusions on heritability. However, the small sample size is taken into account in the confidence intervals and highest posterior density intervals, and hence the results are robust e.g. as to finding that in the common garden there was no population difference similar to that observed in the wild. The low number of offspring in the common garden analysis resulted partly from low survival in the southern population, but although mortality was thus high, there is no reason to suspect that it would have influenced the results. It is also true that the age structure of the wild and common garden data did not overlap, a fact reflected in the difference between the ranges of snout-vent lengths (see Results). Finally, while environmental conditions can be controlled in the laboratory, this also often unavoidably distorts them from natural conditions. For example, while we had two larval temperature treatments, neither of them arguably fully corresponded to the normal temperature conditions common frogs experience in the wild - while temperatures in the experiment were constant, in the wild they fluctuate daily and seasonally and vary spatially. In addition, the temperature treatments were limited to larval stage, ending after metamorphosis. Although these issues should be kept in mind when interpreting our results, the conclusions we have drawn should be justified in the light of the available evidence and information.

## Conclusions

Latitudinal and altitudinal clines in the degree of melanism provide an opportunity to study both the evolution of coloration and evolutionary processes in general. A genetic basis is necessary for any adaptive explanation for such a cline to be applicable. While we observed a positive correlation between latitude and the degree of melanistic pigmentation in the common frog, there was no straightforward genetic foundation for it. This emphasizes the potential role of environment in the degree of melanism, while leaving population demographic explanations and the possible genetic basis of environmental plasticity to be addressed in future studies.

## Authors' contributions

AL, KIJ and JM conducted the field study, and FS and AL the common garden experiment. GH measured the degree of melanism in the wild-caught frogs. JSA conducted the analyses and had primary responsibility of preparing the manuscript, with contributions and comments from all of the other authors.
